# Automatic prostate and prostate zones segmentation of magnetic resonance images using DenseNet-like U-net

**DOI:** 10.1038/s41598-020-71080-0

**Published:** 2020-08-31

**Authors:** Nader Aldoj, Federico Biavati, Florian Michallek, Sebastian Stober, Marc Dewey

**Affiliations:** 1 Department of Radiology, Charité Medical University, Berlin, Germany; 2 Otto-von-Guericke-University Magdeburg, Magdeburg, Germany

**Keywords:** Biomedical engineering, Medical imaging

## Abstract

Magnetic resonance imaging (MRI) provides detailed anatomical images of the prostate and its zones. It has a crucial role for many diagnostic applications. Automatic segmentation such as that of the prostate and prostate zones from MR images facilitates many diagnostic and therapeutic applications. However, the lack of a clear prostate boundary, prostate tissue heterogeneity, and the wide interindividual variety of prostate shapes make this a very challenging task. To address this problem, we propose a new neural network to automatically segment the prostate and its zones. We term this algorithm Dense U-net as it is inspired by the two existing state-of-the-art tools—DenseNet and U-net. We trained the algorithm on 141 patient datasets and tested it on 47 patient datasets using axial T2-weighted images in a four-fold cross-validation fashion. The networks were trained and tested on weakly and accurately annotated masks separately to test the hypothesis that the network can learn even when the labels are not accurate. The network successfully detects the prostate region and segments the gland and its zones. Compared with U-net, the second version of our algorithm, Dense-2 U-net, achieved an average Dice score for the whole prostate of 92.1± 0.8% vs. 90.7 ± 2%, for the central zone of $89.5 \pm 2$% vs. $89.1 \pm 2.2$ %, and for the peripheral zone of 78.1± 2.5% vs. $75 \pm 3$%. Our initial results show Dense-2 U-net to be more accurate than state-of-the-art U-net for automatic segmentation of the prostate and prostate zones.

## Introduction

**Figure 1:**
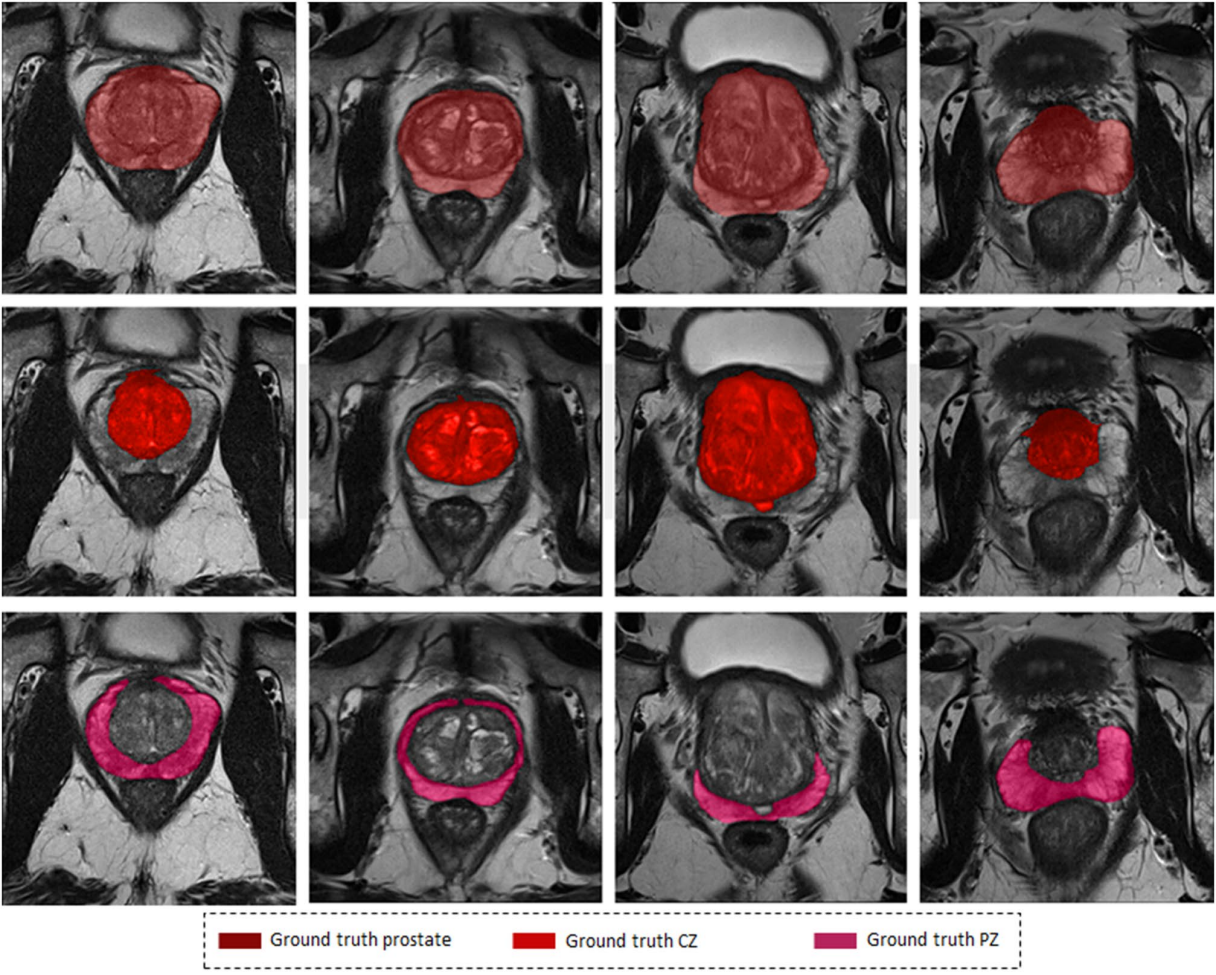
Images illustrating of variations in the MR appearance of the prostate gland in 4 different patients (columns), and rows from top to bottom show Prostate, CZ and PZ respectively.

Prostate cancer (PCa) is the second leading cause of cancer death in the male population^1^. With the massive expansion of prostate screening, PCa has become the most commonly diagnosed cancer in American men^2^. Thus, accurate prostate segmentation has an essential role in many medical imaging and image analysis tasks such cancer detection, patient management, and treatment planning including surgical planning^3^. This involves quantitative volumetric measurements^4–6^. Manual segmentation is still the most common way to accurately segment the prostate gland and prostate regions. However, manual segmentation is a very time-consuming task; furthermore, it is subjective and depends on the level of experience, resulting in poor reproducibility and high interobserver variation. Therefore, reliable automated segmentation of the prostate gland and prostate zones is highly desirable in daily clinical practice. Automated prostate segmentation from magnetic resonance (MR) images is very challenging, for several reasons^7^. First, the ambiguity of its boundaries makes it very hard to differentiate the gland from surrounding tissue with intraprostatic tissue heterogeneity further contributing to under- or oversegmentation. Second, examinations on different MR images with use of different imaging protocols lead to wide variations in signal intensity. Third, the prostate gland has a wide range of sizes, shapes and tissue types, either due to physiologic variations among patients or due to the presence of pathology^8^ see Fig. 1. This is why it is difficult to segment the prostate in general and the peripheral zone in particular.

Many methods and algorithms have been proposed for automated prostate segmentation including atlas-based segmentation^9^, deformable models^10^, spatially max-flow model^11^, and machine-learning-based methods such as random forest, marginal space learning^12^, c-means clustering and zonal morphology^13^, and pattern recognition approach^14^. Currently, deep convolutional neural networks (CNNs) are the dominant and most promising method of automated segmentation for both medical and semantic applications. CNNs are based on the extraction of features in a hierarchical fashion where they have superior performance compared with hand-crafted features. Many studies have investigated CNN-based approaches for medical image segmentation using various modalities. Christ et al.^15^ used a fully convolutional neural network, while Li et al.^16^ used a hybrid densely connected U-net for liver and hepatic tumor segmentation in MR and computed tomography (CT) images. Roth et. al.^17^ used a cascaded neural network with a coarse-to-fine segmentation scheme for multi-organ segmentation ranging from large organs to small vessels. Deniz et. al.^18^ presented a convolutional neural network for proximal femur segmentation, and Kushibar et. al.^19^ investigated the use of neural networks for automatic subcortical brain structure segmentation. Several approaches for automatic prostate segmentation using MR images have been reported. Some of these approaches are based on slice-wise segmentation Zhu et al.^20^ and others on 3D subvolumes (3D patches) segmentation Milletari et al.^8,21^. Few studies have so far addressed the problem of segmenting not only the prostate but also the different zones within the prostate. For instance, Zabihollahy et al. used parallel two U-nets to segment the prostate and its zones on T2w and ADC maps and they achieved a mean Dice score of $92.96 \pm 7.77$ for the best model tested^22^. Clark et al.^23^ developed a network architecture based on U-net^24^ and the inception model^25^, which allows segmentation of the prostate gland and the transitional zone using diffusion-weighted MR images (Dice score of 0.93 and 0.88 for prostate and transitional zone, respectively). Roth et al.^26^ used a 3D U-net to segment the peripheral and transitional zones (Dice scores of 0.85 and 0.60, respectively).

Chilali et al.^27^ proposed an atlas-based and c-means clustering for prostate and zonal segmentation and achieved Dice values of 0.81, 0.70, and 0.62 for the prostate, the transition zone, and peripheral zone, respectively. Tian et. al.^28^ trained and tested a CNN called PSNet to segment the prostate gland on three independent datasets and yielded satisfactory results in terms of Dice score of 85.0±3.8 %. On the other hand, Rundo et al.^29^ incorporated a squeeze-and-excitation module into the state-of-the-art U-net and tested this architecture to segment the prostate and its zones, their method outperformed the other tested state-of-the-art approaches. In a different study, Rundo et. al.^30^ compared different CNN architecture against each other an concluded that U-net outperformed all other methods that were tested. In CNN-based segmentation, the small differences in shape and appearance of prostate glands is usually ignored, Therefore, Karimi et al.^31^ proposed a CNN that incorporates the statistical shape models, their results showed a significant improvements in comparison to the normal CNN approaches. Coarse-to-fine segmentation was used by Jia et. al.^32^ where they used an atlas registration, followed by a CNN-based pixel classification and finally an ensemble learning for fine segmentation, their results showed a superior improvements with Dice value of 0.910±0.036. On the other hand, Cheng et al.^33^ proposed a holistically nested network to segment the prostate gland and claimed to achieve a significantly higher value of Dice score when compared to the patch-based CNN. However, these methods, which use one plane (e.g., axial plane) to perform 2D or 3D segmentation, are limited by low accuracy in the apical and basal area of the prostate. Meyer et. al.^34^ proposed a neural network that takes the three standard planes - coronal, sagital and axial - as input to generate a 3D prostate with segmentation of the central gland, achieving a Dice score of 92.4 and 90 for the prostate and central gland, respectively.

In this work, we present a novel network architecture inspired by U-net^24^ and DenseNet^35^. Our approach combines the strengths of the two networks for segmentation of the prostate gland and its zones. Three variants of the network are tested under the name Dense-*X* U-net, where *X* represents the variant and hence the number of dense blocks. The network is also tested on two variations of segmentation masks, coarsely and fine annotated segmentation masks, to investigate how the variability of ground truth affects segmentation. Briefly, segmentation is done in a slice-wise fashion, and the segmented masks are validated and tested against other state-of-the-art methods such as Classical U-net^24^, cascaded U-net^17^, and PSPNet^36^ using the Dice score, mean relative absolute volume difference (MRAVD), mean Hausdorff distance (MHD), mean surface distance (MSD), sensitivity (Sen) and specificity (Spc).

## Results

We trained all networks on 141 3D volumes (with a total of 2927 axial T2-weighted images) and then tested them on 47 3D volumes (with a total of 980 axial T2-weighted images) from patients in a four-fold cross-validation manner. Two variations (coarse and fine) of segmentations were used to study the effect of ground truth variations to the overall network’s performance. When a coarsely annotated dataset was used, the Dense-2 U-net (Dense U-net with two blocks) achieved an average and median Dice score for the prostate of 91.2±0.8% and 90.3%, respectively,See Appendix for different versions of the Dense U-net. In addition, the Dense-2 U-net had a higher Dice score of 89.2±0.8% for the Central zone (CZ) 76.4±2% for the peripheral zone (PZ) in comparison to the classical U-net with 87.4±1.4% and 74.0±2%, respectively. The results of all statistical measures are compiled in Table 1.

**Table 1:** Statistical analysis of the segmentation results. The Dense-2 and classical U-net blocks. The table presents the average scores across all four-folds obtained when testing the network on the coarsely annotated dataset.

Network	MDS%	CI 95%	StD(%)	MeDS(%)	MRAVD(%)	MHD (mm)
Classical U-net (PR)	89.2	± 0.8	± 3	88.7	44.3	11.7
Dense-2 U-net (PR)	91.2	± 0.8	± 3	90.3	36.1	11.6
Classical U-net (CZ)	87.4	± 1.4	± 5	86.1	15.8	7.5
Dense-2 U-net (CZ)	89.2	± 0.8	± 3	88.1	9.6	7.1
Classical U-net (PZ)	74.0	± 2	± 7	75.0	21.0	8.8
Dense-2 U-net (PZ)	76.4	± 2	± 7	77.2	17.2	9.9

When the second variation of segmentations (finely annotated dataset) were used, all previously tested networks improved in term of overall performance, see Table 2. The Dense-2 U-net had an average and median Dice score for the prostate of 92.1±0.8% and 92.2% compared with 90.7±2% and 92.3% for the classical U-net. In addition, the Dense-2 U-net had a higher Dice score of 89.5±2% for the CZ and 78.1±2.5% for the PZ compared with 89.1±2.2% and 75.0±3%, respectively, for the classical U-net. Furthermore, Table 2 show results of other tested networks such as cascaded U-net which showed inferior performance to the classical U-net and our Dense-2 U-net. PSPNet showed inferior performance to the Dense-2 U-net and superior performance to the classical U-net.

**Table 2:** Statistical analysis of the segmentation results between all tackled networks in this study. The table presents the average scores across all four-folds obtained when testing the network on the revised dataset.

Network	MDS(%)	CI 95(%)	StD(%)	MeDS(%)	MRAVD(%)	MHD (mm)	Sen(%)	Spc(%)
PSPNet (PR)	91.1	± 8	± 3	91.6	37.3	11.6	90.6	99.7
Cascaded U-net (PR)	87.1	± 2	± 7	87.2	39.8	11.2	81.7	99.7
Classical U-net (PR)	90.7	± 2	± 7	92.3	40.7	11.5	87.9	99.8
Dense-2 U-net (PR)	**92.1**	± 0.8	± 3	92.2	41.1	11.3	92.1	99.7
PSPNet (CZ)	89.2	± 1.5	± 5	89.1	7.3	5.8	88.9	99.7
Cascaded U-net (CZ)	85.2	± 2.2	± 7	86.1	5.7	6.6	77.1	99.6
Classical U-net (CZ)	89.1	± 2.2	± 8	88.4	9.9	7.3	83.0	99.7
Dense-2 U-net (CZ)	**89.5**	± 2	± 7	89.4	9.6	6.1	93.9	99.6
PSPNet (PZ)	77.1	± 3	± 10	78.9	22.6	17.6	75.1	99.8
Cascaded U-net (PZ)	71.6	± 2.9	± 10	71.5	22.7	10.5	74.6	99.7
Classical U-net (PZ)	75.0	± 3	± 10	76.5	22.7	10.7	80.1	99.7
Dense-2 U-net (PZ)	**78.1**	± 2.5	± 9	79.5	20.9	20.8	71.7	99.8

Best dice values are in bold.

All numbers (those provided so far and those following) were obtained using cross-entropy as the main loss function, while Table 3 shows a comparison for all three losses (focal, cross-entropy, and Dice) that were used to optimize the network.

**Table 3:** Comparison of dice, focal, and cross-entropy loss and their effect on the performance of the network (Dense-2 U-net).

Loss function	MDS%	StD(%)	MeDS(%)	MHD (mm)
Cross-entropy (PR)	92.1	±3	92.2	11.3
Focal (PR)	91.1	±4	91.2	11.6
Dice (PR)	91.5	±3	91.2	11.5
Dice + Cross-entropy (PR)	92.0	±3.3	92.0	10.3
Dice + Focal + Cross-entropy (PR)	91.7	±3.9	92.1	11.1
Cross-entropy (CZ)	89.5	±7	89.4	6.1
Focal (CZ)	88.5	±5	88.4	7.6
Dice (CZ)	88.2	±5	86.9	7.7
Dice + Cross-entropy (CZ)	89.2	±5	88.9	6.5
Dice + Focal + Cross-entropy (CZ)	88.6	±6	88.6	7.2
Cross-entropy (PZ)	78.1	±9	79.5	20.8
Focal (PZ)	74.4	±11	75.5	17.8
Dice (PZ)	73.4	±11	74.8	19.4
Dice + Cross-entropy (PZ)	78.1	±10	76.8	23.4
Dice + Focal + Cross-entropy (PZ)	77.2	±8.2	76.1	23.7

**Figure 2:**
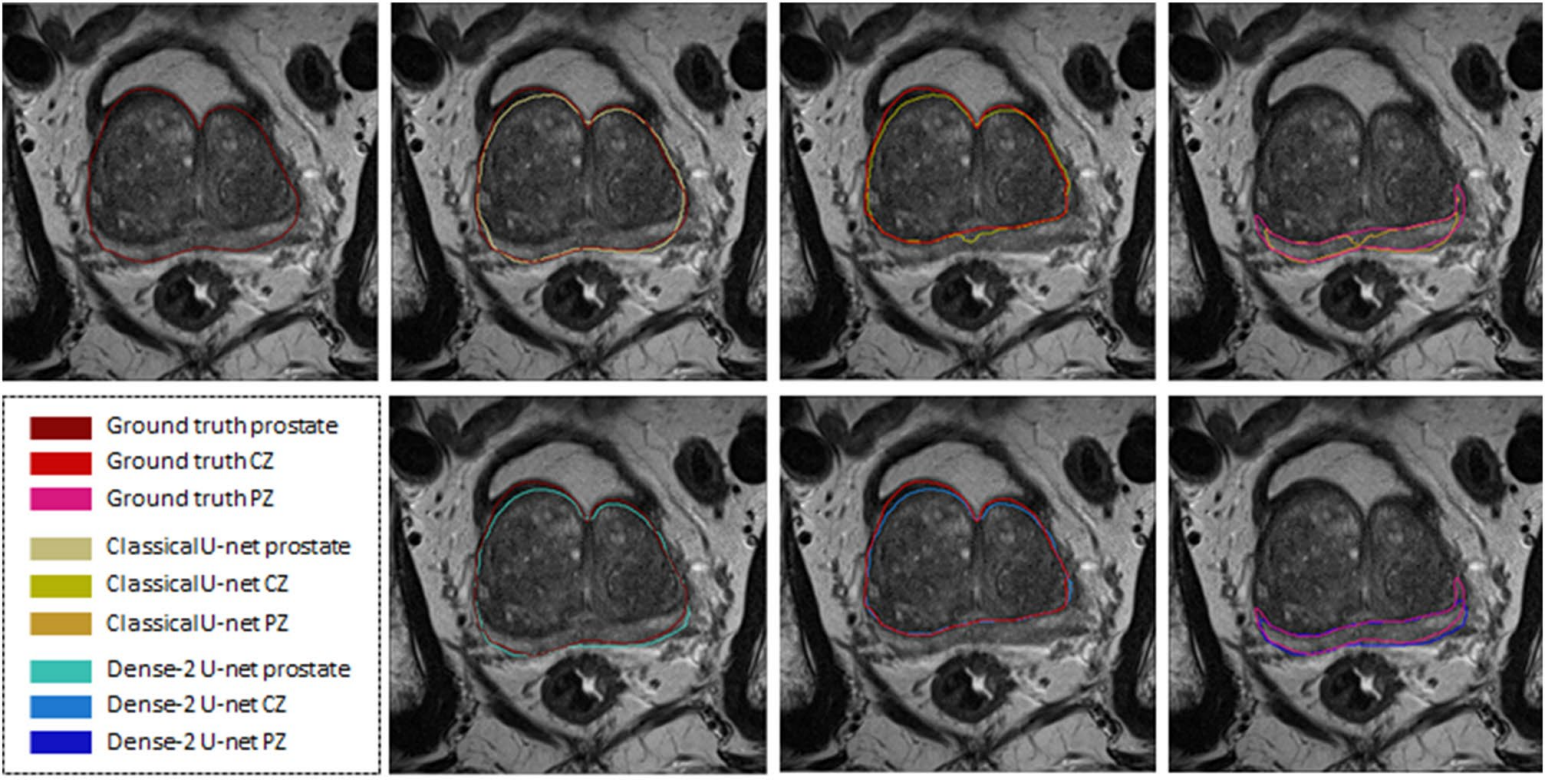
Segmentation results of the classical (first row) and Dense-2 U-net (second row) algorithms. From left to right, ground truth, prostate, CZ and PZ.

**Figure 3:**
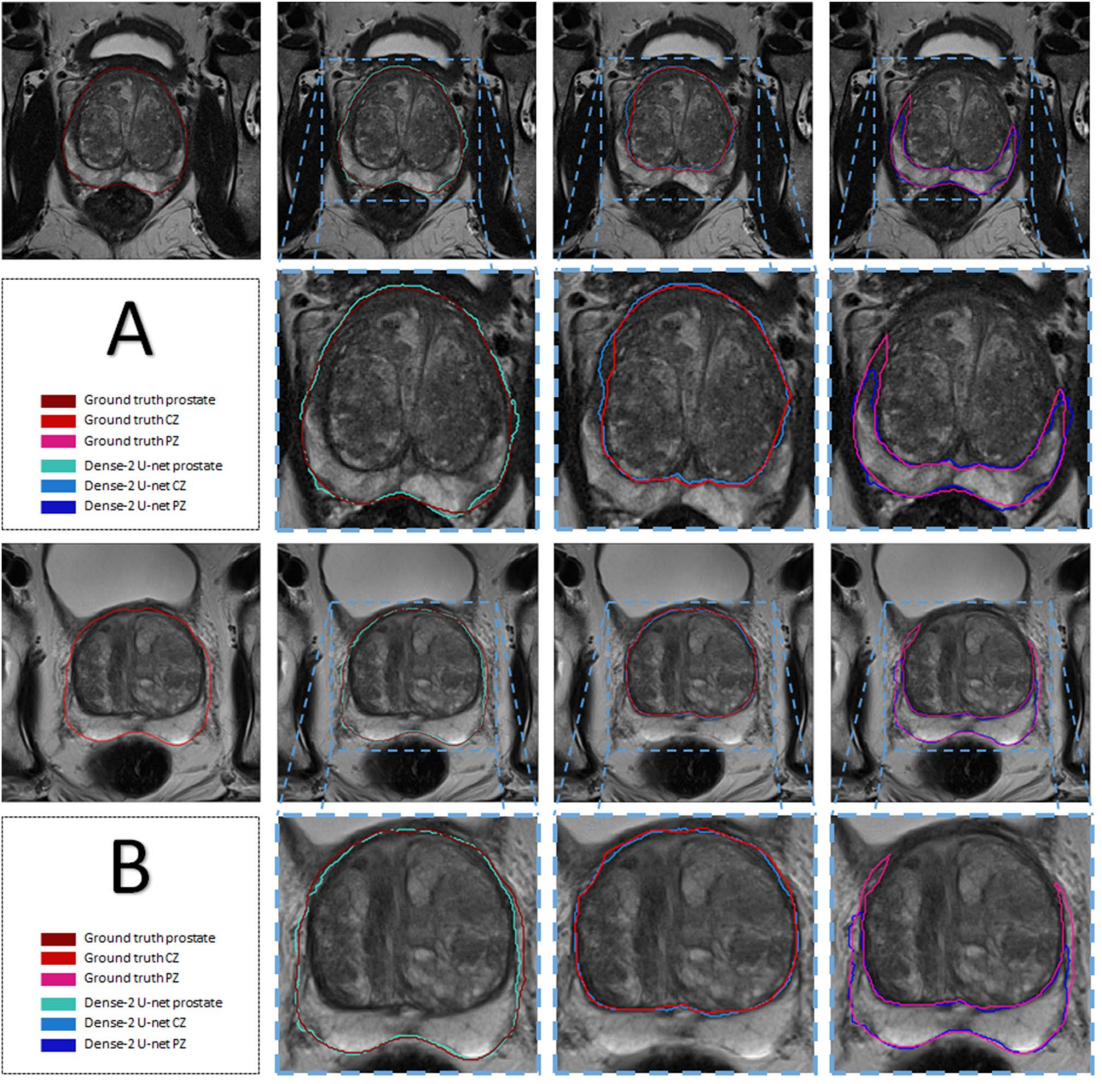
Segmentation of the prostate and its zones (Dense-2 U-net) of two examples (**A**) and (**B**): Columns from left to right show images the original image with the prostate outlines, predicted masks of prostate, CZ and PZ respectively with their corresponding ground truth and an overlay, and a magnification of the overlap; the rows from top to bottom show two examples (**A**) and (**B**)in the 1st and 3rd row and the magnifications on the 2nd and 4th.

**Figure 4:**
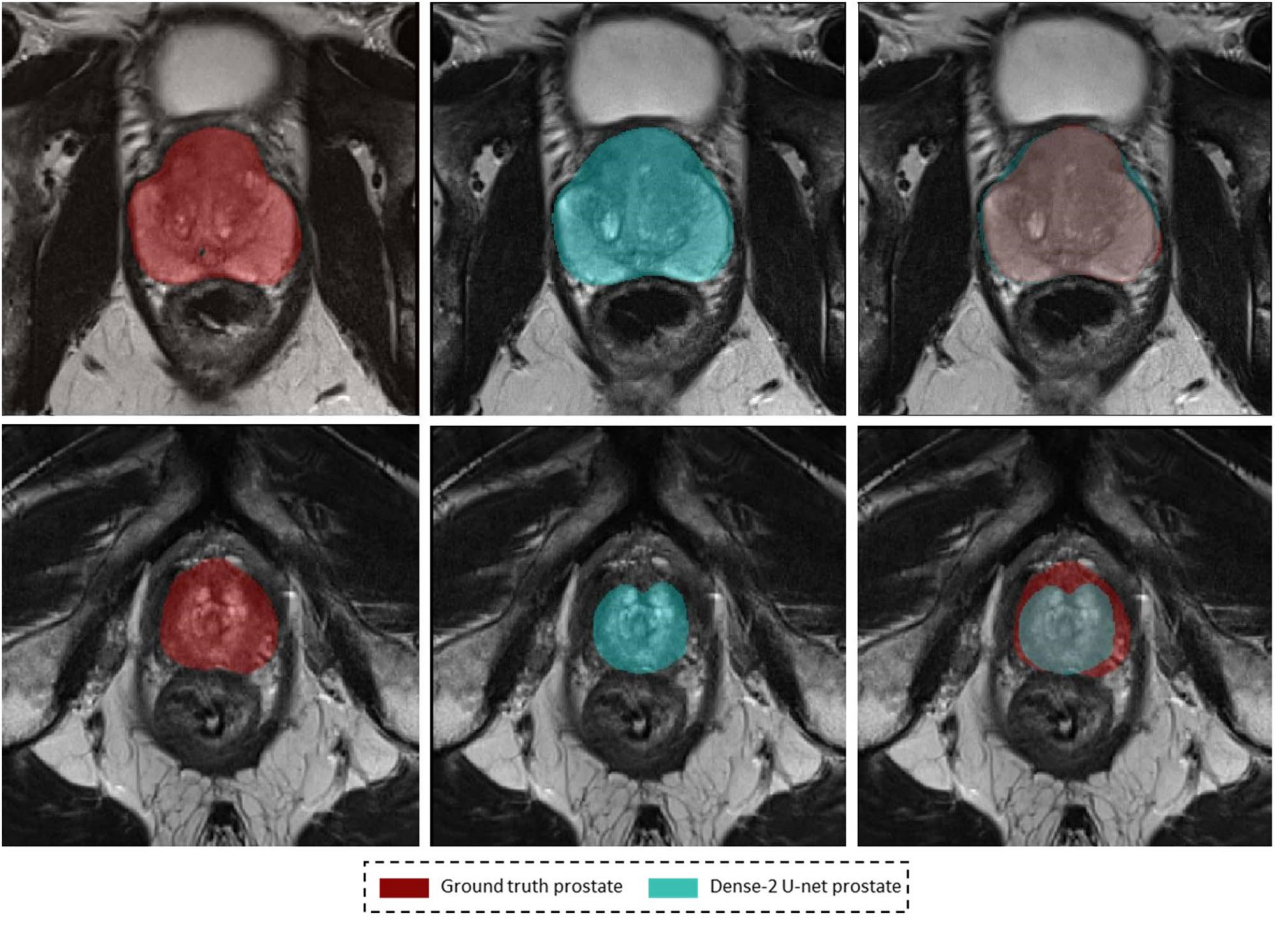
Segmentation results of the Dense-2 U- net: (left) ground truth, (middle) predicted segmentation mask, (right) overlap between ground truth and predicted segmentation mask. The top row shows images of the mid-gland, and the bottom row shows images of the apex.

As we can see from Table 2, all networks performed accurate segmentation of the prostate gland and its zones with some examples for illustration shown in Figs. 2 and 3. However, it is also apparent that for segmentation of the middle region, a relatively higher average Dice score of 94% was obtained for the Dense-2 U-net and 93% for classical U-net in comparison to a relatively lower value for the apical and basal regions of the prostate (Dense-2 U-net: 72% and 80%; Classical U-net: 71% and 77%), respectively; see Fig.  4.

**Figure 5:**
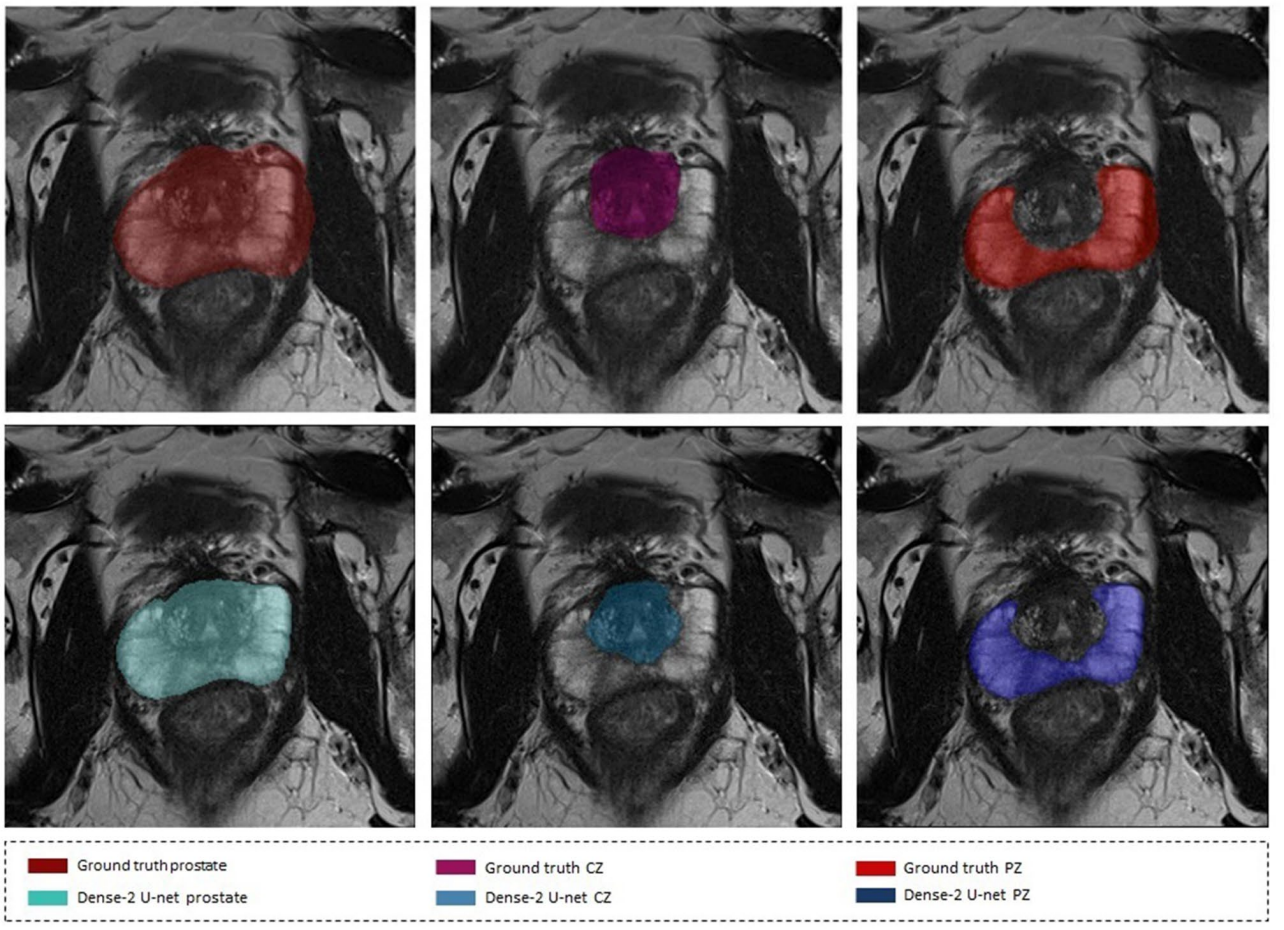
Segmentation results on a weakly annotated dataset. The upper row shows the weakly annotated ground truth while the bottom row shows the accurate prediction of the network (Dense-2 U-net) for prostate, PZ, and CZ from left to right.

Figure 5, shows the segmentation of the network against the coarsely annotated labels.

**Figure 6:**
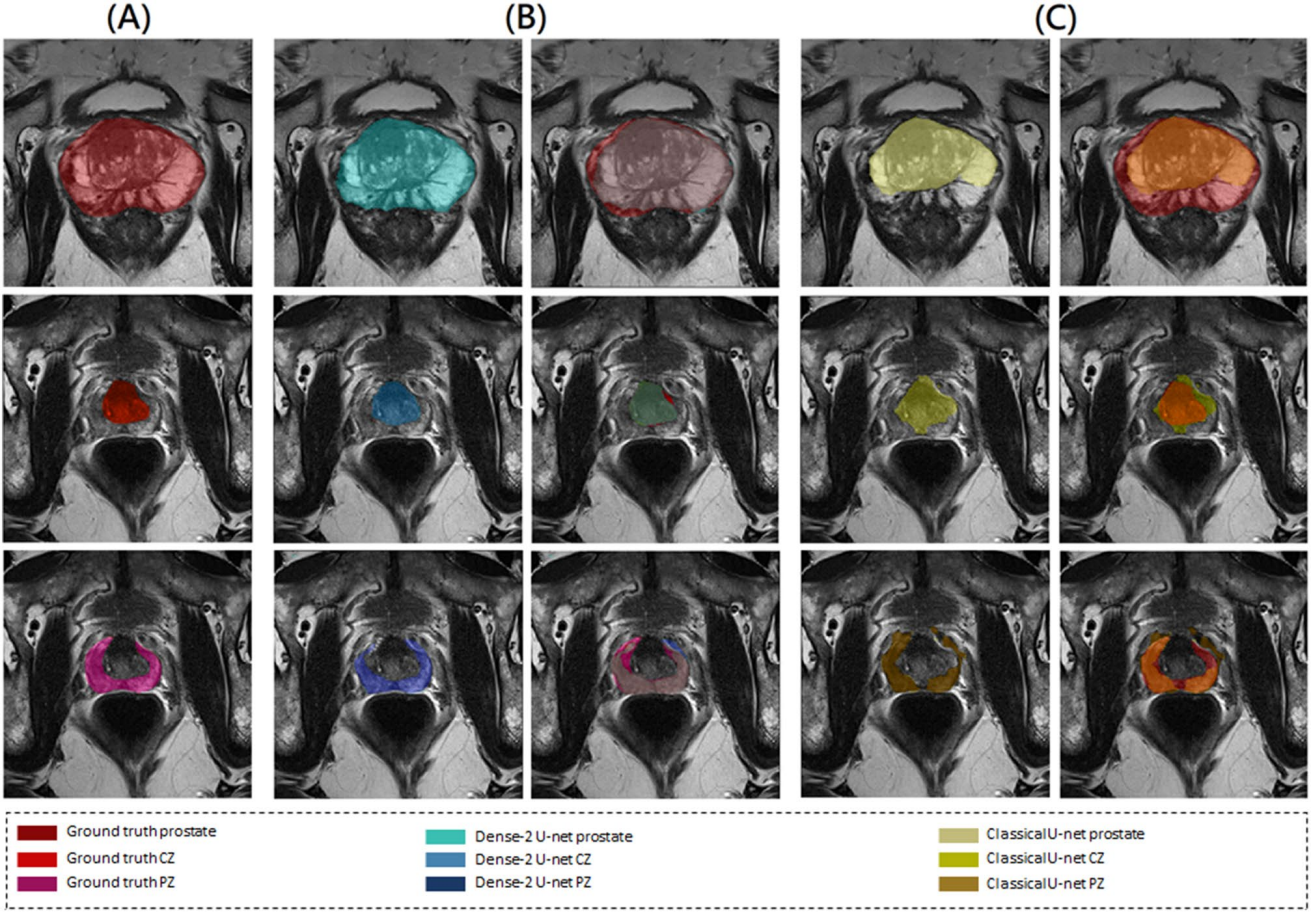
Ground truth images (**A**), segmentation results of Dense-2 (**B**) and the classical U-net (**C**) (first row) for the prostate, (second row) for PZ, and (third row) for CZ, where in each of the subfigure, ground truth is on the left side, predicted mask is in the middle and the overlap is on the right side.

Table 4 presents the results of statistical analysis for both Dense-2 and classical U-net for the four-fold cross-validation, showing that even though Dense-2 U-net was better than the classical U-net, this difference was not statistically significant in the first three cross-validation folds, while it was significant on the fourth fold. Figure 6 shows some examples illustrating significantly better segmentation of Dense-2 U-net compared with the classical U-net.

We tested both networks (Dense-2 and classical U-net) using rigid and elastic augmentation separately and a combination of the two augmentation methods. Table 6 compiles the results for each of the augmentation methods and their effect on the Dice score of each network.

**Table 4:** Statistical significance of four-fold cross-validation $p<0.05$ according to the *t* test applied to scores of Dense-2 and classical U-net

	1st fold	*p* value	2nd fold	*p* value	3rd fold	*p* value	4th fold	*p* value	Average	*p* value
PR Dense-2	92.3	0.30	92.2	0.38	91.6	0.29	92.0	**0.00001**	92.1	0.17
PR Classic	92.2		92.4		91.3		87.1		90.7	
CZ Dense-2	88.6	0.41	89.8	0.1	89.0	0.06	90.0	**0.00001**	89.5	0.34
CZ Classic	89.1		90.9		88.9		87.5		89.1	
PZ Dense-2	78.5	0.39	78.4	0.3	76.4	0.45	78.9	**0.003**	78.1	0.17
PZ Classic	77.3		76.3		79.9		66.3		75.0	

where significant *p* values are marked in bold.

Regarding visual inspection for both networks, visual Dice scores were (mean±standard deviation) 0.844±0.034 for classical U-net, 0.851±0.03 for Dense-2 U-net , 0.861±0.03 for human reader for whole-prostate segmentation and 0.743±0.024 for classical U-net, 0.745±0.025 for Dense-2 U-net , 0.758±0.025 for human reader for peripheral zone segmentation. Differences for neither whole-prostate nor peripheral zone-only segmentations were statistically significant.

**Figure 7:**
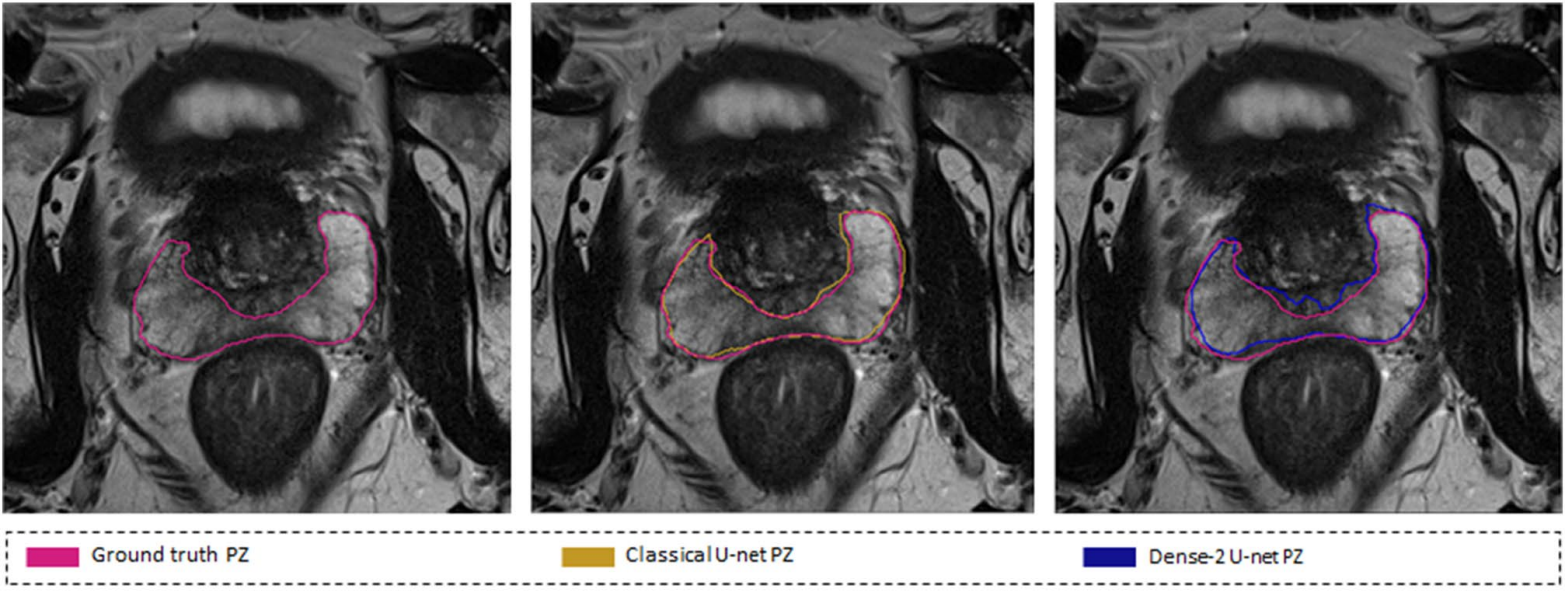
Contour consistency: (left) ground truth, (middle) overlap between the classical U-net segmentation mask and the ground truth, and (right) overlap between Dense-2 U-net segmentation mask and the ground truth.

Mean Hausdorff distance was computed to measure the distance between the predicted prostate delineation and the ground truth. It is apparent, see Fig. 7, that the classical U-net provided superior contour consistency to the Dense-2 U-net in PZ, and approximately similar contour consistency in the prostate and CZ, see Table 2.

**Figure 8:**
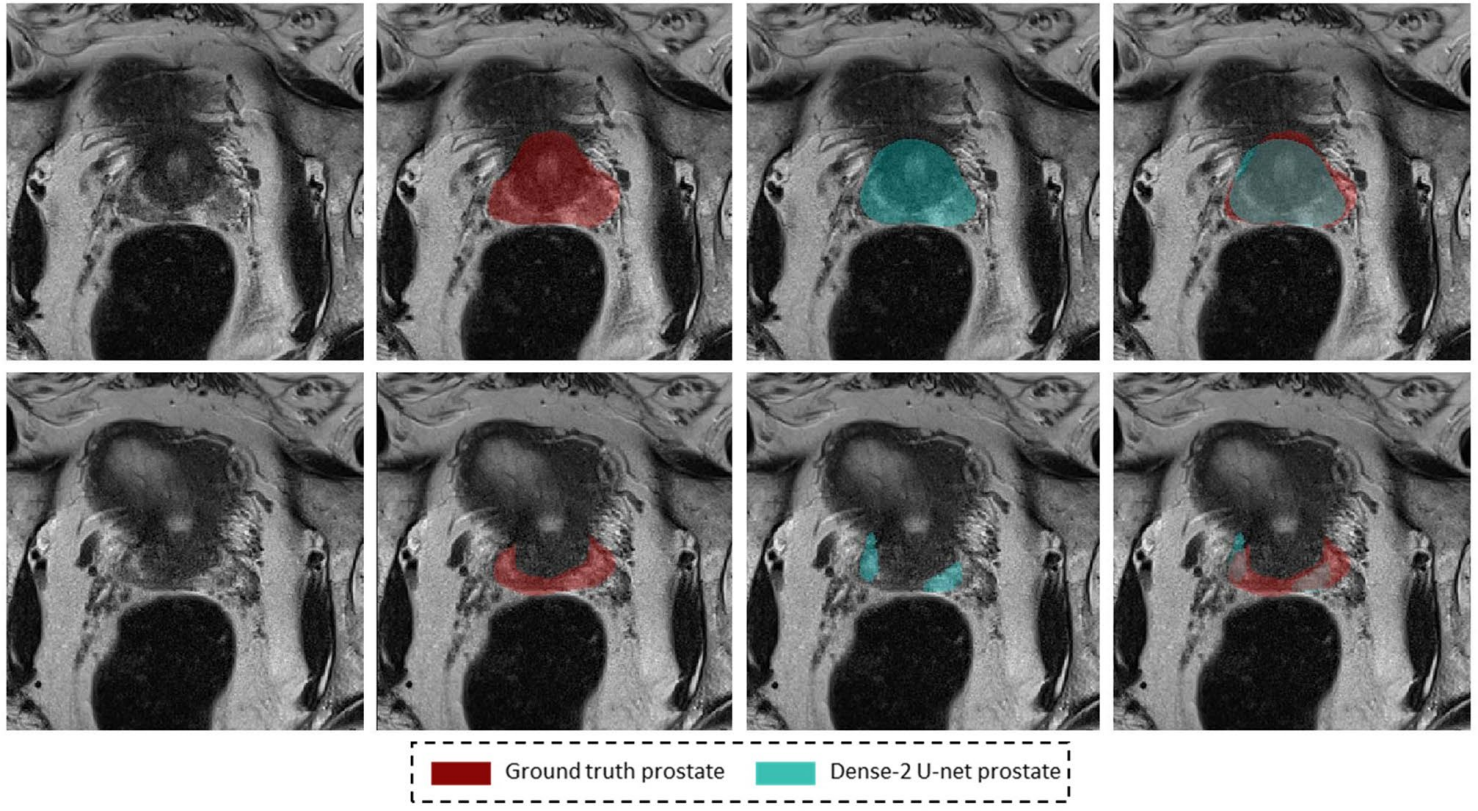
Examples of inaccurate segmentation: the first row shows examples of inaccurate segmentation of the prostate gland while the second row shows examples for the peripheral zone.

**Figure 9:**
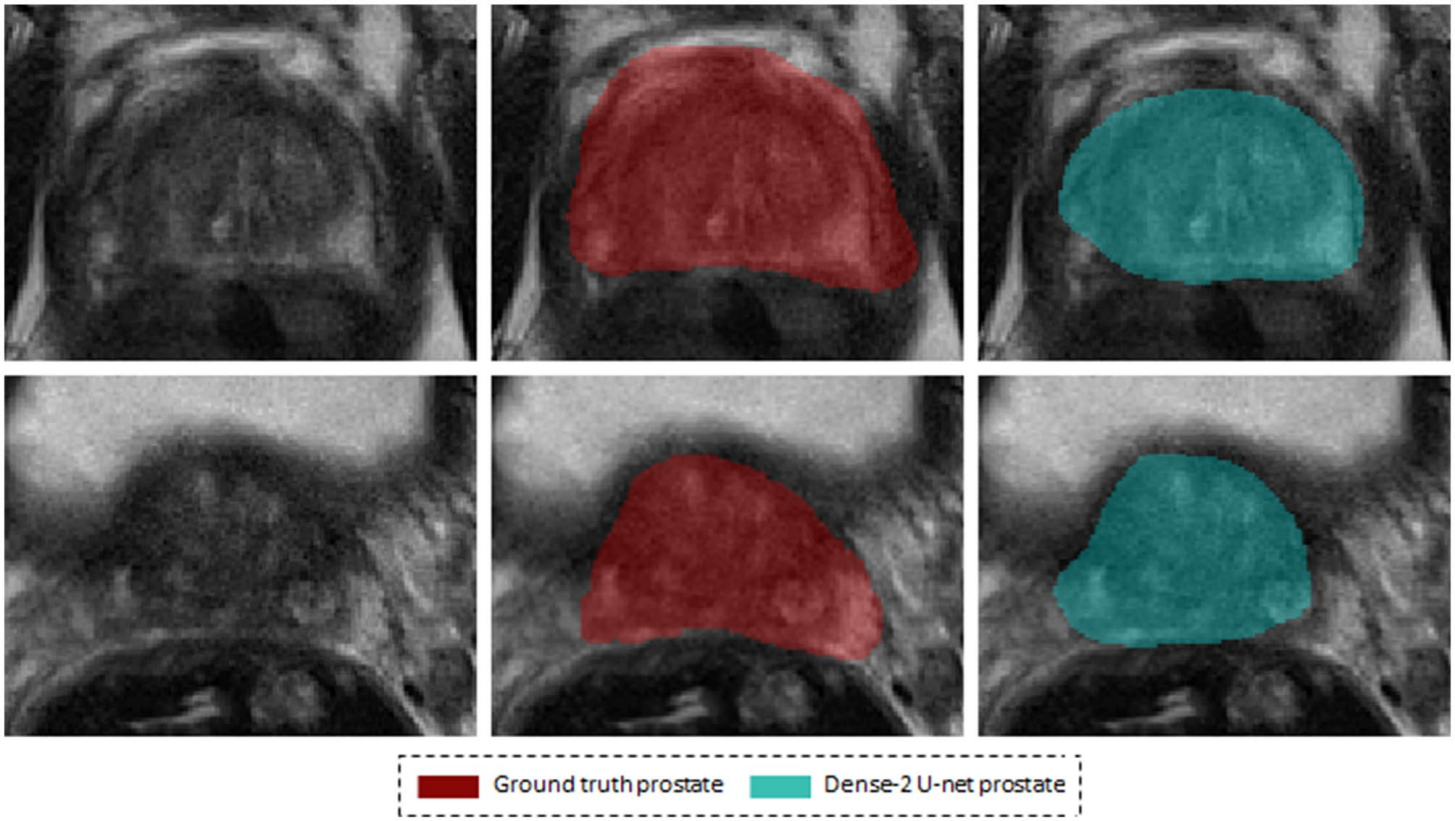
Motion artifacts and their negative effect on segmentation. The images in the center are the ground truth and the images on the right are the result of segmentation with the Dense-2 U-net.

Some slices were not accurately segmented by any network; examples of failure to recognize the prostate border or that of the peripheral zone are presented in Figs. 8 and 9.

## Discussion

The prostate gland usually has fuzzy boundaries, and pixel intensities are heterogeneous both inside and outside the prostate. Furthermore, contrasts and pixel intensities are very similar for prostate and non-prostate regions. All of these factors taken together make prostate segmentation a very challenging task. Nevertheless, Fig. 2 shows that both classical and Dense-2 U-net allow accurate segmentation of the prostate and its zones. However, in our investigation, Dense-2 U-net achieved a higher average Dice score for the prostate of 92.1±0.8% compared with 90.7±2% for the classical U-net. In addition, the Dense-2 U-net had a higher Dice score of 89.5±2% for CZ and of 78.1±2.5% for PZ compared to 89.1±2.2% and 75.0±3% for the classical U-net. Compared to all other methods mentioned in this study, See Table 2, Dense-2 U-net achieved slightly higher scores than all tested methods such as cascaded U-net and PSPNet. This improved performance is attributable to the nature of the Dense-2 U-net, which is based on feature map concatenation, which means that one convolutional stage has direct access to all previous feature maps from all successive stages. This enables feature map reuse. Furthermore, concatenating feature maps from different stages enhances input variations and makes data flow through the model more efficient. As the role of the transitional layer in our network is to ensure a homogeneous number of feature maps inside and at the end of each stage, it also helps in compressing feature maps and making the model more compressed. Dense-2 U-net is more efficient in terms of training time (discussed later), and more compact especially when compared to other models mentioned previously such as cascaded U-net^17^ or parallel U-nets^22^ where the need of two networks in series or parallel is a necessity.

Intraobserver variability was calculated for a single radiologist segmenting the images in different time points, while interobserver variability was calculated for different radiologists; the results are presented in Table 5. We can see that Dense-2 U-net achieved a slightly higher Dice score of 92.1±0.8% than the single radiologist 90.0±0.8%. This indicates that the network achieved a very high accuracy close to the performance of a radiologist.

**Table 5:** Intra- and inter-observer variability.

Observer variability	MDS (%)	CI 95 (%)	StD (%)	MeDS (%)	MRAVD (%)	MHD (mm)	MSD (mm)
Intra-(prostate)	90.4	± 1.1	± 4	91.7	12.8	20.31	2.92
Intra-(peripheral zone)	79.2	± 2.5	± 9	82.2	16.6	13.72	1.15
Intra-(central zone)	82.7	± 3.3	± 12	87.5	11.8	10.79	1.15
Inter-(prostate)	86.6	± 1.4	± 5	87.4	14.9	22.82	2.98
Inter-(peripheral zone)	75.7	± 1.6	± 6	80.0	17.8	25.27	1.51
Inter-(central zone)	83.5	± 1.4	± 5	85.3	12.3	17.17	1.5

The visual Dice scores obtained here signify excellent performance of all three networks (Classical, and Dense-2 U-net) investigated. Results are very close to the ground truth provided by the human reader with a slightly better performance of Dense-2 U-net over the normal U-net.

Figure 5, which compares the segmented masks and the coarsely annotated labels shows that the network learned accurate delineation of the prostate gland and its zones although some of the labels which the network was trained on were coarsely annotated. We can thus conclude that the network produces better predictions than available in the datasets on which it was initially trained, provided that enough accurate examples are presented to the network during training^37^.

While the classical U-net employs only long skip connections between opposite stages of the encoder and decoder part, which are beneficial for training very deep networks and facilitate the flow of the gradient, Dense U-nets use short skip connections between the different convolutional layers at each stage, which helps in stabilizing parameter updates^38^. The combination of long and short skip connection improves overall network performance.

The Dense-2 U-net showed noticeably better performance, suggesting that the improvement may be mainly attributable to the second block. On the one hand, we may argue that deeper networks (Dense-2 U-net) perform better because they can have more abstracted features. On the other hand, Dense-2 U-net extracts more features and has more short skip connections, resulting in smoother and easier training since they facilitate the flow of the gradient and stabilize the parameter update. Additional variants of Dense U-net were tested and results can be seen in the Appendix.

As we can see from Table 4, Dense-2 U-net was more accurate in all cross-validation folds; yet the difference in performance was not significant in the first three fold with *p* values of 0.3, 0.38, and 0.29 for the first, second, and third fold, respectively. However, in the fourth fold, the network showed a statistically significant improvement in performance with *p* values of 0.00001, 0.00001, and 0.003 for prostate, PZ, and CZ, respectively, compared to the classical U-net. Images illustrating this improvement are presented in Fig. 6. The results suggest that Dense-2 U-net can learn more difficult cases than the classical U-net especially with regard to structural discontinuities or border ambiguity. Figure 6 panel (c). The first row shows examples of areas missed in segmentation by the classical U-net due to tissue heterogeneity and discontinuity or abrupt changes in gray values. The second and third rows show oversegmented masks of the PZ and CZ. Here, the network was not able to follow the borders of the prostate zones, resulting in discontinuous, fuzzy, and inaccurate masks.

**Figure 10:**
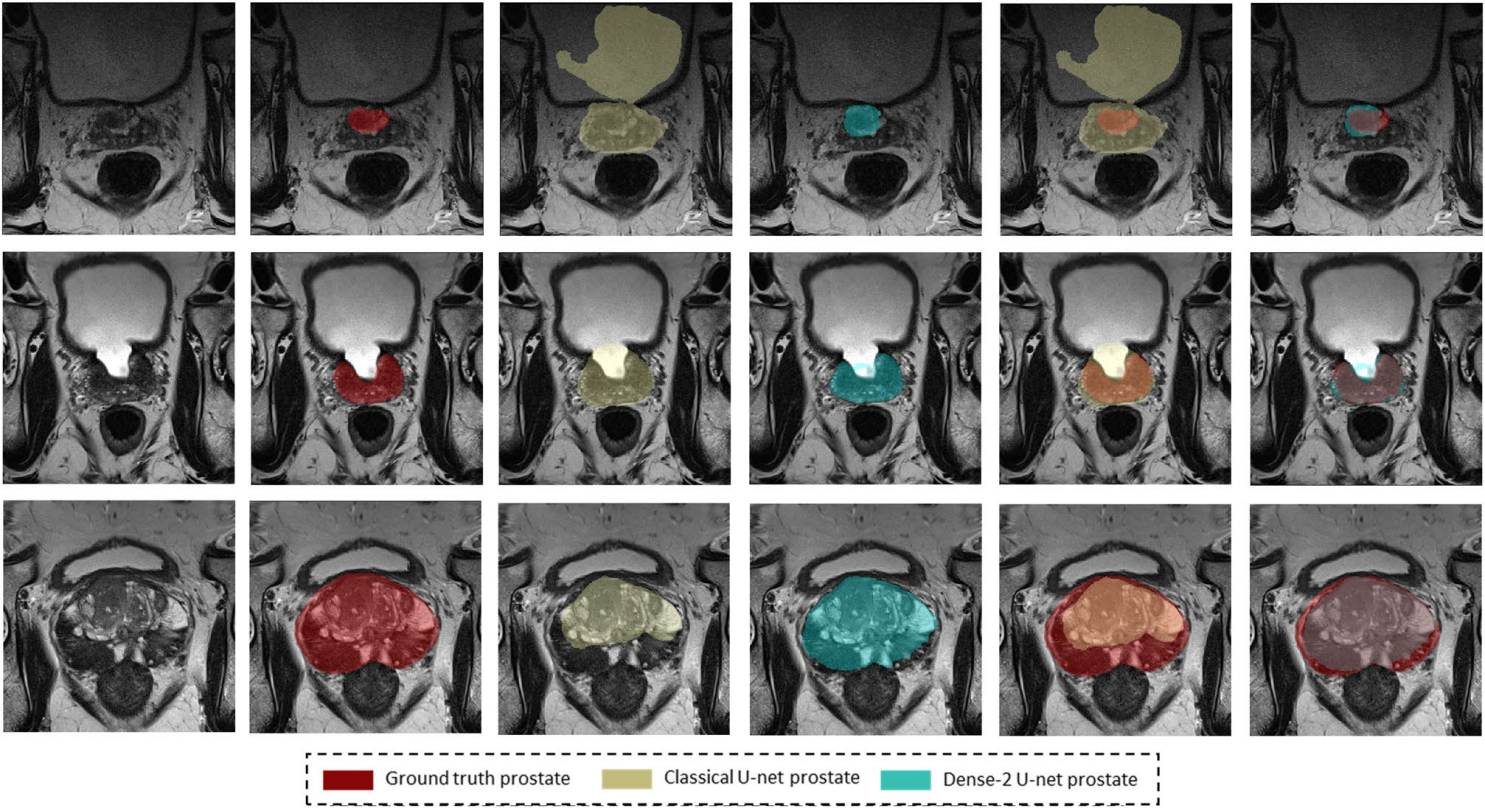
Images illustrating several special cases in the dataset. Each raw represents a different case. Red masks indicate the ground truth segmentations, while yellow and blue masks represent the masks generated from classical and Dense-2 U-net respectively, and the last two columns show the overlap between ground truth and predict masks of the aforementioned networks.

Figure 10 shows also that this fold contained several cases that the classical U-net was inaccurate in segmenting the prostate and its zones. On the other hand, the Dense-2 U-net was able to cope with those difficult cases and achieved better accuracy in segmenting the wanted regions. Below we explain three cases that they were special in this test set: The first patient (see Fig.  10 first row) resembles the case where a benign prostatic hyperplasia (BPH) grows into the bladder. In this case, the classical U-net could not discriminate between the bladder and the prostate tissue and hence, a big part of the bladder was mis-segmented by the classical U-net as a prostate. On the other hand, Dense-2 U-net was able to discriminate between the two tissue types and kept the border lines as close as possible to the ground truth. The second patient (see Fig. 10 second row) was a Transurethral resection of the prostate (TURP) where the patient undergoes a surgery to shave the excess prostatic tissue that blocks the urinary track due to the enlarged prostate. In this case, there is a void inside the prostate where it looks like a bladder, and this should not be mistaken and segmented as a prostate tissue. The classical U-net segmented the whole area as prostate including the void which was caused by TURP, while the Dense-2 U-net was sensitive to this area and did not include it in the prostate segmentation, that would explain why Dense-2 U-net did better in terms of Dice score. The third patient (see Fig.  10 third row) was a case where a big tumor was on either side of the prostate. The classical U-net did not consider the cancerous tissue as part of the prostate, which is wrong since this tissue is still prostate even though that the tissue characteristics are different, yet it should still be segmented as prostate. The Dense-2 U-net was able to segment this cancerous tissue as part of the prostate, some of the edges were still missed, however, the major part of the tumor was included in the final segmentation. The above mentioned results showed that, although the reported difference between Dice scores of the classical and Dense-2 U-net does not seem to be significant as a quantitative value, the qualitative results shown in the images testify that the Dense-2 U-net was more reliable when it comes to special and difficult cases.

Inaccurately segmented cases are presented in Fig.  8. The first row shows an example of prostate undersegmentation compared with the ground truth, while the second row shows an example of discontinuous segmentation of the peripheral zone. This inaccuracy could be due to many factors, especially tissue heterogeneity, partial volume effects, and border ambiguity play, which make segmentation very challenging. Furthermore, a radiologist usually scrolls through the volume to see multiple slices and seeks help from a previous or later slice(s) in order to perform a segmentation on a slice where the prostate or the zone is not well defined. This option is not available to a network since it performs segmentation in a slice-wise fashion without access to 3D information. Moreover, 3D networks do not necessarily perform better especially due to the large partial volume effect as observed in^38^. Motion artifacts can markedly degrade segmentation accuracy by both human readers and networks as illustrated in Fig. 9. Here, the prostate borders are blurred and merged with surrounding tissues, making the prostate’s edges indistinguishable and difficult to follow.

In terms of convergence, Dense-2 U-net has faster convergence and achieved the best performance after 35,760 training iterations while the classical U-net performed best after 36,952 training iterations. This result implies that Dense-2 U-net is easier to train due to the fact that it has many concatenation connections enabling the reuse of feature maps and facilitating data flow throughout the network. This, in turn, results in faster convergence.

All networks investigated here require roughly the same time for training (around 20 h for the classical U-net and 18 h for Dense-2 U-net) and inference (2 s for one test 3D volume).

We chose to split the volume in this order 25% apex, 50% mid-gland and 25% base to represent the different part of the prostate gland. As seen in Fig. 4, the network performs more reliably in segmenting the middle region of the prostate gland than the apical and basal peripheral regions. Various factors may make it harder to segment the apical and basal regions such as greater ambiguity of the prostate border, partial volume effects, tissue heterogeneity, and the lower number of representative slices in comparison to the mid-gland. This limitation might be overcome by augmenting the last few slices of the upper and lower prostate and training the network on the whole set with the augmented slices so that the network will not be biased towards the higher number of slices that represent the middle region. This approach was implemented and tested but did not result in any noticeable improvement in terms of the overall Dice score.

Table 3 shows results of Dense-2 U-net with different losses. Cross-entropy loss gave the best performance, which is consistent with published data showing that using different loss functions did not improve the results significantly^38,39^. Focal loss showed lower performance than cross-entropy and a slightly better performance than the Dice loss. Combining both Dice and Cross-entropy losses, and all the losses were investigated and reported in the Table 3 and it can be seen that there is no improvements over the plain Cross-entropy loss. Therefore, cross-entropy loss was used for further network training and testing at all stages of the study.

Hausdorff distance was computed to investigate the contour consistency of the predicted masks. It is obvious, see Fig.  7, that the classical U-net yielded a better contour consistency than the Dense-2 U-net for PZ and roughly similar values in the prostate and CZ see Table 2. One can conclude, see Table 2, that the resulting Dice score of the Dense-2 U-net is higher than the classical U-net, yet the border’s delineation of the PZ using the classical U-net is much more reliable. Additionally, when considering MRAVD values, we see that there is no big difference between the classical and Dense-2 U-net in both prostate and CZ and a small improvement on the PZ segmentation in the Dense-2 compared to the classical U-net.

Looking at Table 6, we can clearly observe that neither of the two types of augmentation (rigid and elastic) had no positive effect on network performance, which is consistent with the results in^39^. This could be due to the fact that the networks did not face an overfitting problem that could be solved by an augmentation process. Moreover, the number of images (slices) that was provided to the network during training was not small enough to cause overfitting. Possibly, the augmentation methods that were used here did not really capture the variability of the images in the test set and thus did not add any improvement to the overall performance of the network. Use of image augmentation resulted in five times longer training time for both networks than the original time without any augmentation.

**Table 6:** Statistical analysis of augmentation.

	Dense-2 U-net			Classical U-net		
	Dice score(%)	StD(%)	Median (%)	Dice score (%)	StD (%)	Median (%)
Prostate						
No aug.	92.1	± 3	92.2	90.7	± 7	92.3
Elastic	92.2	± 2	92.6	90.0	± 3	89.7
Rigid	92.6	± 2	92.1	89.3	± 4	88.9
Both	92.5	± 3	92.1	92.6	± 3	82.5
**PZ**						
No aug.	78.1	± 9	79.5	75	± 2	76.5
Elastic	77.9	± 9	77.0	76.1	± 8	77.9
Rigid	77.1	± 10	76.8	75.1	± 10	76.2
Both	78.2	± 10	77.4	80.5	± 10	80.9
**CZ**						
No aug.	89.5	± 7	89.4	89.1	± 8	88.4
Elastic	89.6	± 4	89.0	87.7	± 8	87.2
Rigid	90.4	± 3	89.9	86.9	± 7	85.3
Both	90.7	± 3	89.6	90.1	± 4	89.6

In summary, the performance of all networks investigated here is reliable and accurate with a level similar to that of radiologists. While the performance difference between the classical and Dense-2 U-net is not significant, the Dense-2 U-net achieved a better MDS in general yet an inferior MHD in PZ, hence a less consistent contour delineation in PZ. Despite that the reported difference between Dice scores of the classical and Dense-2 U-net does not seem to be significant as a quantitative value, the qualitative results shown and discussed in the study testify the Dense-2 U-net was more accurate in segmenting special and difficult cases. The qualitative improvements presented above were subjectively appreciated by our radiologists even though there resulted in quantitative improvements that were not significant. Finally, neither the change in architecture, nor the loss function or data augmentation led to any significant improvement in overall network accuracy.

## Methods

### Problem formulation

Given an image *I* with dimensions *N* and *M*, segmentation can be considered a partitioning of the image into adjacent segments *S* with a distinct label {*0, .. K*} for each.

$$\begin{aligned} \bigcup _{k=0}^{K} r_k=I, \quad \text {for} \quad r_k \subseteq {I} \end{aligned}$$

The segmentation process will output a labeled image *L(i,j)* in which each pixel has a distinct value in the range of {*0, .. K*}.

$$\begin{aligned} L(i,j) = S ; \quad for \quad L(i,j) \in \{0,K\} \quad and \quad \forall (i,j)\in S \end{aligned}$$

*L(i,j)=0* represents the background and *L(i,j)=d* for *d=1,..,K* determines the region of interest, i.e., the prostate gland and its zones.

### Patient data

In this study, a dataset of T2-weighted MR images of 188 patients was used (public dataset, PROSTATEx challenge from Radboud University)^40–43^. T2- weighted images were acquired on 3T MR Siemens scanners (MAGNETOM Trio and Skyra) with a turbo spin echo sequence with 0.5 mm in-plane resolution and 3.6 mm slice thickness. All images were segmented manually and interpreted by an experienced radiologist. Coarsely and accurately segmented images were included in both training and test sets to investigate the hypothesis of whether or not the networks can learn accurate segmentation from coarsely annotated images and correct for weakly annotated segmentations. The weakly annotated images were resegmented to produce accurate labels, and all networks were retrained and retested on the new accurate labels. We used 141 patients (including a total of 2982 slices) as the training set and 47 patients (including a total of 912 slices) as the test set, on which all networks were trained and tested in four-fold cross-validation fashion. All images were first resampled to a common resolution of 0.5, 0.5 mm in x, y direction. Then the images were cropped with a 256x256 pixel window positioned at the center of the 2D image. Image normalization was done on the fly during training and testing of the network.

### Image augmentation

**Figure 11:**
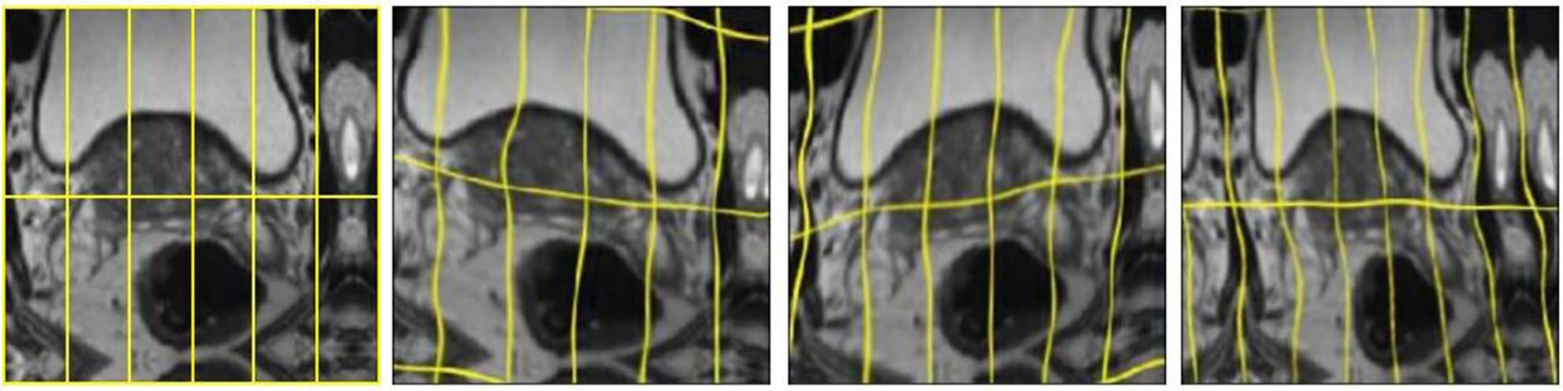
Image augmentation using elastic deformation with different parameters values.

Image augmentation was done on the flight using two methods: elastic and rigid transformation. Five random elastic image deformations and four rigid transformations (flipping, rotation, zooming, and translation) were used. Elastic deformation as described in^44^ is controlled by tow parameters, the elasticity coefficient $\sigma$, which was set to 512, and a scaling factor $\alpha$, which controls the intensity of the deformation and was set to 21. Examples are provided in Fig.  11. The two augmentation methods were tested against each other and against both networks (U-net and Dense-2 U-net) without augmentation.

### U-net

The architecture of the U-net^24^ simply consists of two parts, an encoding (compression) and a decoding (decompression) part with skip connections in between mirroring stages. Each stage consists of a number of convolutional operations with a specific kernel size, normally 3x3, and followed by a rectified linear unit (ReLU)^45^. At the end of each stage in the encoding part, a max pooling operation with a stride of 2 is used for downsampling the resulting feature maps. In the decoding part, an upsampling operation with a stride of 2 is used to gradually increase the dimension of the feature maps until the full image size is reached at the end of the network. With every convolution operation, some information will be lost. Thus, skip connections between the encoding and the decoding part are important to compensate for this loss and help to ease the flow of information throughout the network layers and hence speed up convergence.

### Dense U-net

**Figure 12:**
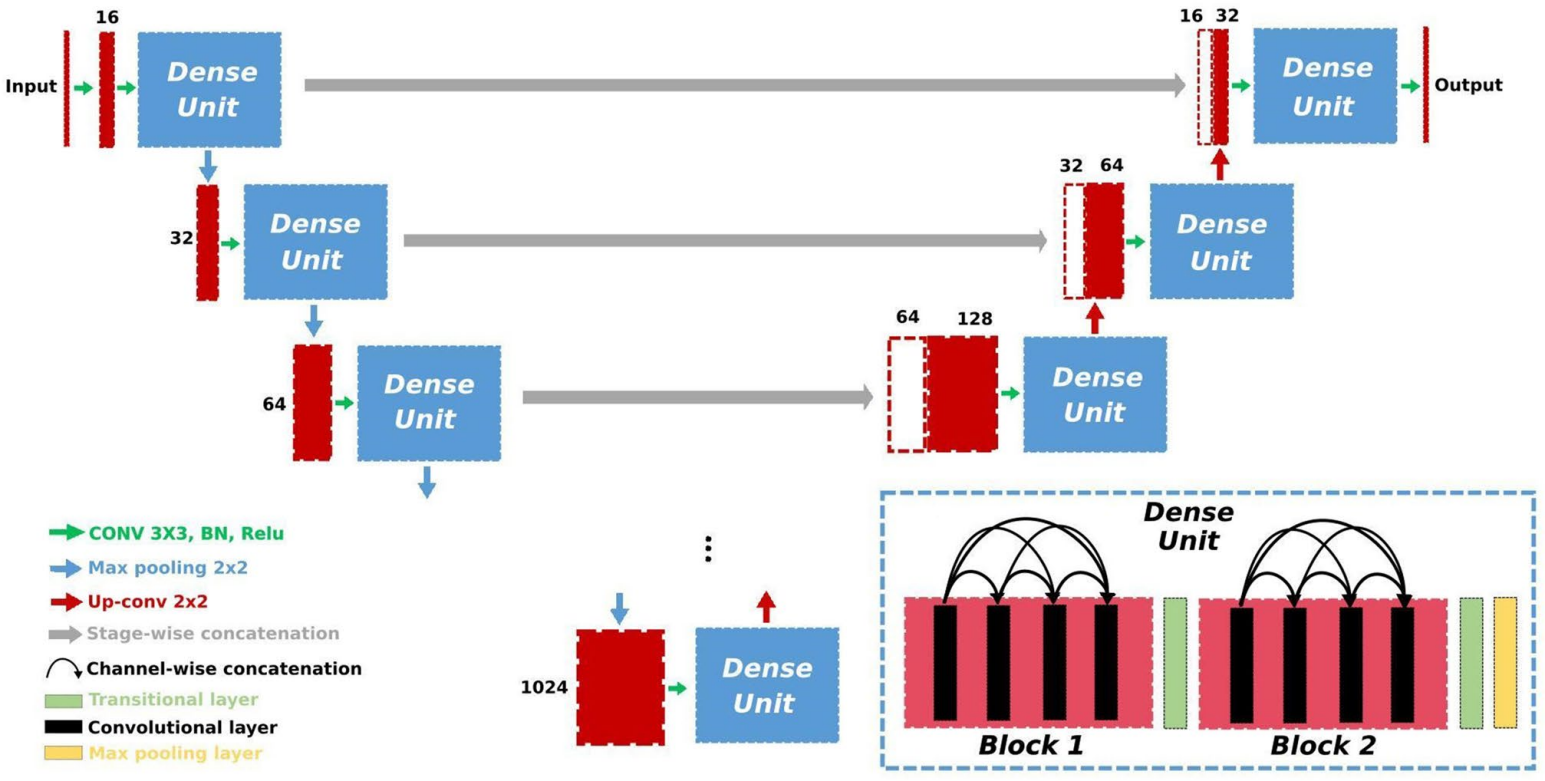
The Dense-2 U-net architecture. Numbers in the figure indicate the number of feature maps at each stage.

The architecture of the new Dense-2 U-net we propose here is depicted in Fig. 12. Three variations of the network are studied, (named Dense-*X* U-net, where *X* represents the variant and hence the number of dense blocks) where a combination of four convolutional and one transitional layers followed by an identical combination is used in every Dense block. The depth of the network is very crucial for accuracy^46^; therefore, the deeper the network, the better the results. However, a deeper network means a higher number of parameters, which in turn makes the network more prone to overfitting, especially in medical imaging, where the number of available examples is limited. Furthermore, deeper networks require more computational resources. Dense U-net, in general, is based on the U-net architecture with 6 stages in the encoding and the decoding part. We replaced the normal stack of convolutional layers with a DenseNet-like architecture, which could consist of one (Dense-1 U-net), two (Dense-2 U-net) or three (Dense-3 U-net) small dense blocks separated by transitional layers. Each of the dense blocks comprises 4 convolutional layers with a kernel size of 3x3 and is followed by a ReLU. Their input is the concatenated output from all respective previous layers within the block, which in turn helps in retaining some of the information lost because of the convolutional operations. Due to channel-wise concatenation, the number of feature maps might vary in each block; thus, a transitional layer is introduced after the Dense block to ensure that the resulting feature maps are always the same as in the desired output of each stage. This layer also plays a role as a compressing layer that reduces the dimensionality and the number of parameters needed for each stage. The dense blocks used in the encoding part have the same architecture as the ones in the decoding part. In contrast to the encoding part of the network, which uses max pooling with a stride 2 for downsampling, the decoding part in Dense U-net uses an up-convolutional operation with a stride 2 for upsampling, as was recommended by^47^. Focal cross-entropy or Dice loss^48^ with AdamOptimizer^49^ were used to train the network.

Both Classical and Dense-2 U-net shared an architecture of six stages in depth and feature maps of 16 up to 1024 at the bottleneck stages with an increment rate of 2 to the power of 4 up to 10. Kernels of sizes 3x3 and stride of 2 at the end of each stage were used. While the total number of parameters of the classical U-net was 33 million, the Dense-2 U-net that we developed in this study had 15.6 million, which was less than half the number of parameters of the classical U-net. Transition layers at the end of each dense block played a role in homogenizing the number the feature maps that were concatenated from different convolutional layers inside the dense block which in turn helped in the model compactness.

### Comparison

The comparison in our study was done between several variations and versions of the Dense U-net (Dense-2 is reported in the main text and other versions can be seen in the appendix) and other state-of-the-art methods. In summary, a classical U-net was used as the basis and one of the state-of-the-art, cascaded version of U-net was tested following the approach presented by^17^, a U-net was used as a backbone for the pyramid scene parsing network (PSPNet) approach^36^.

### Loss functions

The behavior of the neural network is highly dependent on the choice of the loss function due to the fact that the network will learn and update its parameters according to the partial derivative values with respect to the learned weights. Hence, it is important to choose the right loss function which drives the optimization process toward the desired end. For training our neural network, we compared the behavior of the network with three different loss functions: cross-entropy, focal^48^, and Dice loss^8^. Cross-entropy loss is given by the following formula:

$$\begin{aligned} CE(a,b) = - (a log (b) + (1-a) log(1-b))&\end{aligned}$$

where *a* is the prediction mask and *b* the ground truth, and performs pixel-wise comparison between the predicted mask and the ground truth. Focal loss is basically a modified version of cross-entropy loss with addition of two variables which control down-weight easy examples in favor of the hard ones and it is represented as follows:

$$\begin{aligned} FL(a,b) = - (\alpha (1-b)^\gamma a log (b) + (1-a)b^\gamma log(1-b)) \end{aligned}$$

where $\alpha$ and $\gamma$ are used for the class imbalance and the focus on hard examples . The Dice coefficient, on the other hand, is presented as a loss function since it is the main evaluation term regarding segmentation, which makes it the most reasonable term to be used as a derived of the network optimization procedure. It is given as:

$$\begin{aligned} DL(a,b) = 1- \frac{2\sum ab}{\sum a + \sum b}&\end{aligned}$$

where *a* is the predicted mask and *b* the ground truth image.

### Evaluation

Manual delineations of the more experienced radiologist were used as the ground truth to evaluate the performance of the networks. We tested the Dense-2 U-net (See appendix for more comparisons) against the classical U-net and evaluated segmentations by calculating the mean Dice score (MDS) with 95% confidence interval (CI), median Dice scores (MeDS), standard deviation (Std), mean relative absolute volume difference (MRAVD), mean Hausdorff distance (MHD) as a contour consistency measure, mean surface distance (MSD) and *t* test with $p<0.05$ as a statistical significance measure.

The Dice score (DS)^50^, also known as F1-score or the similarity coefficient, measures the overlap between the ground truth and the predicted segmentation mask. It is widely used for evaluating segmentation volumes in medical images^51^. For two binary sets *A* and *B*, DS is the ratio of the intersection to average cardinality.

$$\begin{aligned} DS(A,B) = \frac{2|A \cap B|}{|A| + |B|} \end{aligned}$$

The relative absolute volume difference is calculated by taking the division between the total volume of the resulted mask and the volume of the ground truth. From this number 1 is subtracted and the result is multiplied by 100 and expressed as percentage.

$$\begin{aligned} RAVD(A,B) = \left( \frac{B}{A} -1 \right) *100 \end{aligned}$$

The Hausdorff distance is the maximal distance from a point in the first mask (the predicted segmentation) i$\in$ A to its nearest point in the ground truth j$\in$ B and is defined as follows

$$\begin{aligned} HD(A, B) = max_{i\in A} \{ min_{j\in B} \parallel I-J \parallel \}&\end{aligned}$$

where $\parallel$ I-J $\parallel$ is any norm e.g. the Euclidean distance between two point sets *A* and *B* and is expressed in millimeters (mm)^52^.

For visual evaluation of the segmentation performance of the Dense-2 and classical U-net, an experienced radiologist reviewed the segmentations in random order and assigned a score between 0 and 100 (which we term visual Dice score). This radiologist was not involved in manual segmentation and blinded to all information regarding the segmentation process, specifically, whether the segmentation was performed by either of the two CNNs or by a human reader. Visual Dice scores were evaluated on segmentation of whole-prostate and peripheral zone-only individually. Mean and standard deviation of visual Dice scores were calculated.

### Implementation details

All networks were trained end-to-end on axial T2-weighted MR images. All images had a size of 256x256 and a resolution of 0.5x0.5 mm in x and y directions. Image processing was done using the SimpleITK library, and the network was implemented using Tensorflow 1.4.0. We chose 5 images as a batch size for both networks. All experiments were done using TitanXP with 12 GB of video memory and CUDA version 8.0. The training time of the classical U-net was around 8.5 h while the time for the Dense-2 U-net was around 18.5 h. The computation time during training for a single 3D volume (of around 20 slices) was approximately 2.96 and 6.3 s for classical U-net and Dense-2 U-net, respectively. During testing the computation time for a 3D volume was 0.4 and 0.66 s for classical U-net and Dense-2 U-net, respectively.

### Ethical standard

All the procedures were confirmed and approved by Ethics Committee of Radboud university. All methods were carried out in accordance with relevant guidelines and regulations. The need for informed consent was waived by the local ethics committee of the Radboud University Medical Center.

## Supplementary information

Supplementary Information 1

## Data Availability

All images used in this study are publicly available while the segmented masks were done in house
