# Supplementary material for: Automatic prostate and prostate zones segmentation of magnetic resonance images using DenseNet-like U-net
Source: Sci Rep. 2020 Aug 31;10:14315. doi: 10.1038/s41598-020-71080-0 (PMC7459118; doi:10.1038/s41598-020-71080-0)
Supplement: Supplementary file 1 — Supplementary Information 1 [file 41598_2020_71080_MOESM1_ESM.pdf]

Appendix

Additional variants of Dense U-net were tested in this study (Dense-1 and Dense-3). In case of coarsely annotated dataset, the Dense-2 U-net (Dense U-net with two blocks) achieved an average and median Dice score for the prostate of 91.2±0.8% and 90.3%, respectively, versus 89.3±0.8% and 89.1% with the Dense-1 U-net (Dense U-net with one block), which was higher compared with the classical U-net with 89.2±0.8% and 88.7%, respectively. In addition, the Dense-2 U-net had a higher Dice score of 89.2±0.8% for the Central zone (CZ) 76.4±2% for the peripheral zone (PZ) versus Dense-1 U-net with 87.4±0.3% for the CZ and 74.5±2% for the in comparison to the classical U-net with 87.4±1.4% and 74.0±2%, respectively . The results of all statistical measures are compiled in Table 1.

Table 1: Statistical analysis of the segmentation results. The number in the name (e.g. Dense-2) refers to the number of dense blocks, PR indicates prostate, CZ for central zone, and PZ for peripheral zone. The table presents the results obtained for the training and testing of the network on the weakly annotated dataset

**Network**	**MDS%**	**CI 95%**	**StD(%)**	**MeDS(%)**	**MRAVD(%)**	**MHD (mm)**
Classical U-net (PR)	89.2	±0.8	±3	88.7	44.3	11.7
Dense-1 U-net (PR)	89.3	±0.8	±3	89.1	45.5	11.5
Dense-2 U-net (PR)	91.2	±0.8	±3	90.3	36.1	11.6
Classical U-net (CZ)	87.4	±1.4	±5	86.1	15.8	7.5
Dense-1 U-net (CZ)	87.4	±0.3	±1	87.1	10.1	7.1
Dense-2 U-net (CZ)	89.2	±0.8	±3	88.1	9.6	7.1
Classical U-net (PZ)	74.0	±2	±7	75.0	21.0	8.8
Dense-1 U-net (PZ)	74.5	±2	±7	75.2	24.3	10.0
Dense-2 U-net (PZ)	76.4	±2	±7	77.2	17.2	9.9

Table 2 shows results between all addressed networks together with different variant of Dense U-net after the segmentation masks were revised.

Table 2: Statistical analysis of the segmentation results between all tackled networks in this study. The number in the name (e.g. Dense-2) refers to the number of dense blocks. The table presents the results obtained for the training and testing of the network on the revised dataset

**Network**	**MDS(%)**	**CI 95(%)**	**StD(%)**	**MeDS(%)**	**MRAVD(%)**	**MHD (mm)**	**Sen(%)**	**Spc(%)**
PSPNet (PR)	91.1	±8	±3	91.6	37.3	11.6	90.6	99.7
Cascaded U-net (PR)	87.1	±2	±7	87.2	39.8	11.2	81.7	99.7
Classical U-net (PR)	90.7	±2	±7	92.3	40.7	11.5	87.9	99.8
Dense-1 U-net (PR)	90.5	±1	±4	90.5	40.7	10.8	88.6	99.7
Dense-2 U-net (PR)	**92.1**	±0.8	±3	92.2	41.1	11.3	92.1	99.7
Dense-3 U-net (PR)	91.0	±1.1	±4	91.1	36.6	15.8	92.1	99.6
PSPNet (CZ)	89.2	±1.5	±5	89.1	7.3	5.8	88.9	99.7
Cascaded U-net (CZ)	85.2	±2.2	±7	86.1	5.7	6.6	77.1	99.6
Classical U-net (CZ)	89.1	±2.2	±8	88.4	9.9	7.3	83.0	99.7
Dense-1 U-net (CZ)	88.1	±2.2	±8	87.8	13.3	6.8	84.6	99.7
Dense-2 U-net (CZ)	**89.5**	±2	±7	89.4	9.6	6.1	93.9	99.6
Dense-3 U-net (CZ)	89.5	±1.4	±5	88.8	12.5	5.7	88.3	99.7
PSPNet (PZ)	77.1	±3	±10	78.9	22.6	17.6	75.1	99.8
Cascaded U-net (PZ)	71.6	±2.9	±10	71.5	22.7	10.5	74.6	99.7
Classical U-net (PZ)	75.0	±3	±10	76.5	22.7	10.7	80.1	99.7
Dense-1 U-net (PZ)	73.3	±2.5	±9	75.6	29.8	12.6	78.1	99.6
Dense-2 U-net (PZ)	**78.1**	±2.5	±9	79.5	20.9	20.8	71.7	99.8
Dense-3 U-net (PZ)	76.0	±2.3	±8	76.3	24.9	10.1	81.8	99.6

When the second variation of segmentations (finely annotated dataset) were used, all previously tested networks improved in term of overall performance, see Table 2. The Dense-2 U-net had an average and median Dice score for the prostate of 92.1±0.8% and 92.2% compared with 90.5±1% and 90.5% for Dense-1

U-net and 90.7±2% and 92.3% for the classical U-net. In addition, the Dense-2 U-net had a higher Dice score of 89.5±2% for the CZ and 78.1±2.5% for the PZ compared with 88.1±2.2% for CZ and 73.3±2.5% for PZ with Dense-1 U-net versus 89.1±2.2% and 75.0±3%, respectively, for the classical U-net. Furthermore, Table 2 show results of other tested networks such as cascaded U-net which showed inferior performance to the classical U-net and our Dense-2 U-net. PSPNet showed inferior performance to the Dense-2 U-net and superior performance to the classical U-net. Additionally, Dense-3 U-net (a 3-variant of the Dense U-net where additional block is added) showed intermediate performance between the classical and Dense-2 U-net.

From studying the values presented in Table 1 and 2 , We can thus conclude that the network produces better predictions than available in the datasets on which it was initially trained, provided that enough accurate examples are presented to the network during training.

While Dense-1 U-net was similar to the classical U-net, the Dense-2 U-net showed a noticeably better performance, suggesting that the improvement may be mainly attributable to the second block. Dense-2 U-net extracts more features and has more short skip connections, resulting in smoother and easier training since they facilitate the flow of the gradient and stabilize the parameter update. On the one hand, we may argue that deeper networks (Dense-3 U-net) perform better because they can have more abstracted features, a third variant of the Dense U-net (Dense-3 U-net) was examined and showed intermediate performance between the classical and Dense-2 U-net and hence, In general, deeper networks result in a better performance e.x. ResNet152 is better performing than ResNet101 [1]. However, deeper networks are sometimes harder to train on specific datasets especially when the dataset is small so that the number of data points is less than the parameters of the model and in this case the network is prone to overfitting and may suffer a vanishing gradient, so deeper networks do not always lead to better performance [2]. The same applies to Dense-3 U-net, it is deeper and has more parameters than Dense-2, yet deeper network does not always lead to better results.

It is worth noting that Dice score is an overlapping measurement while the Hausdorff distance is the maximal distance between two points sets. Those two metrics are intrinsically different and there is no correlation between their outcomes. The Dice score could reach a very good value such as 92-99% (depends on the application), yet Hausdorff distance could be big due to that few pixels were misclassified since it is a measurement of a maximal distance. Hausdorff distance could be an indication of contour constancy but not a measurement of accurate segmentation since few misclassified pixels could result in a large Hausdorff distance.

References

[1] Kaiming He, Xiangyu Zhang, Shaoqing Ren, and Jian Sun. Deep residual learning for image recognition. In *Proceedings of the IEEE conference on computer vision and pattern recognition*, pages 770–778, 2016.

[2] Shizhao Sun, Wei Chen, Liwei Wang, Xiaoguang Liu, and Tie-Yan Liu. On the depth of deep neural networks: A theoretical view. In *Thirtieth AAAI Conference on Artificial Intelligence*, 2016.
